# Accuracy of soft tissue balancing in total knee arthroplasty using surgeon-defined assessment versus a gap-balancer or electronic sensor

**DOI:** 10.1186/s13018-021-02439-w

**Published:** 2021-05-08

**Authors:** Ran Zhao, Yanqing Liu, Hua Tian

**Affiliations:** Peking University Third Hospital, Department of Orthopaedics, Engineering Research Center of Bone and Joint Precision Medicine, Ministry of Education, 49 North Garden Road, Beijing, 100191 Haidian District China

**Keywords:** Total knee arthroplasty, Sensor, Gap-balancer, Learning curve

## Abstract

**Background:**

Soft tissue balancing is essential for the success of total knee arthroplasty (TKA) and is mainly dependent on surgeon-defined assessment (SDA) or a gap-balancer (GB). However, an electronic sensor has been developed to objectively measure the gap pressure. This study aimed to evaluate the accuracy of soft tissue balancing using SDA and GB compared with a sensor.

**Methods:**

Forty-eight patients undergoing TKA (60 knees) were prospectively enrolled. Soft tissue balancing was sequentially performed using SDA, a GB, and an electronic sensor. We compared the SDA, GB, and sensor data to calculate the sensitivity, specificity, and accuracy at 0°, 45°, 90°, and 120° flexion. Cumulative summation (CUSUM) analysis was performed to assess the surgeon’s performance during the sensor introductory phase.

**Results:**

The sensitivity of SDA was 63.3%, 68.3%, 80.0%, and 80.0% at 0°, 45°, 90°, and 120°, respectively. The accuracy of the GB compared with sensor data was 76.7% and 71.7% at 0° and 90°, respectively.

Cohen’s kappa coefficient for the accuracy of the GB was 0.406 at 0° (moderate agreement) and 0.227 at 90° (fair agreement). The CUSUM 0° line achieved good prior performance at case 45, CUSUM 90° and 120° showed a trend toward good prior performance, while CUSUM 45° reached poor prior performance at case 8.

**Conclusion:**

SDA was a poor predictor of knee balance. GB improved the accuracy of soft tissue balancing, but was still less accurate than the sensor, particularly for unbalanced knees. SDA improved with ongoing use of the sensor, except at 45° flexion.

## Introduction

Total knee arthroplasty (TKA) is an effective treatment for severe knee diseases, and soft tissue balance is a key factor in achieving a successful outcome [1, 2]. Improper soft tissue balance may result in pain, stiffness, instability, or even polyethylene wear, leading to revision knee surgery [2–4]. Balancing is often determined using surgeon-defined assessment (SDA), which depends on operative experience and lacks an objective and quantitative standard. A gap-balancer (GB) is used to calculate the difference between the internal and external space. However, the variation between surgeons in the strength that they use to extend the gap may result in measurement errors. Furthermore, the distraction gap measurement is only accurate to 1 mm, and can only be measured at 0° and 90° flexion [5].

Sensor can objectively measure the intercompartmental loads of the knee during TKA, previous study compared sensor as a reference to SDA during soft tissue balancing [6]. SDA is reportedly a poor predictor of soft tissue balance compared with the sensor and is even worse at larger knee flexion angles [6]. The purpose of this study was to (1) evaluate the compartment loads throughout the knee range of motion in the sensor-balanced group and determine the sensitivity of SDA; (2) evaluate the sensitivity, specificity, and accuracy of the GB compared with the sensor; (3) verify that SDA of knee balance improves with the use of a sensor.

## Material and methods

### Study cohort

With the approval of the institutional ethics committee, data were prospectively collected from patients who underwent unilateral or bilateral TKA in our hospital from December 2018 to December 2019. Sensors were used in all cases. All patients provided written informed consent for study participation. The inclusion criteria were (1) osteoarthritis; (2) knee varus or valgus of less than 25° and flexion contracture of less than 25°. The exclusion criteria were (1) rheumatoid arthritis, traumatic osteoarthritis, or prior osteotomy around the knee; (2) joint replacement with severe varum/varus/flexion contracture or bone deformity that required an allograft, constrained implants, or mental augmentation.

A total of 48 patients (60 knees) were included (31 left knees and 29 right knees). The average patient age was 66 years (range, 56–76 years), and the average varus deformity angle was 12.6° (range, 5–25°). The Genesis II cruciate-retaining implant (Smith & Nephew, London, UK) was used for all operations. All surgeries were performed by the same senior knee arthroplasty surgeon, who performs approximately 300 to 350 knee surgeries per year and had not used the sensor technology prior to these 60 surgeries.

### Surgical technique

A medial parapatellar approach was used for all surgeries. The deep part of the medial collateral ligament was retained and turned over the patella. The femoral resection was done using an intramedullary guide, while the tibial resection was done using an extramedullary guide to restore a neutral mechanical axis of the lower limb. Standard cutting blocks were used to complete the femoral and tibial preparations, and a spacer was used to guide the soft tissue balancing. A standard trial articular insert was used to determine the appropriate insert thickness. SDA was performed with the joint in four positions: 0°, 45°, 90°, and 120°of flexion. The test was standardized by imparting varus and valgus stress on the knee, with one hand applying maximal stress to the tibia and the other hand stabilizing the femur. During SDA, the balance of collateral ligament tension was determined by visual and tactile assessments of the medial and lateral openings. Soft tissue release was carried out until the balance was considered satisfactory.

**Fig. 1:**
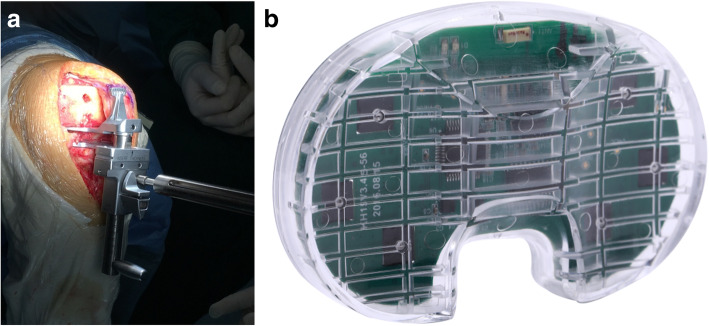
**a** The gap-balancer for the Genesis II prosthesis. **b** The knee joint pressure sensor with a shape that conforms to the Genesis II prosthesis

After the SDA was performed, the balance was checked using a GB and a wireless electronic pressure sensor insert with the same thickness and size (Yiemed Co. Ltd., Shandong, China) (Fig. 1). A GB with 20 lb of force was used to open the knee joint space, and the difference between the medial and lateral compartments was measured at 0° and 90° of knee flexion. The sensor data were recorded with the limb held in neutral alignment to prevent the weight of the limb from stressing or distracting the joint. The sensor was used to determine the pressure in the medial and lateral components at knee flexion angles of 0°, 45°, 90°, and 120°. The data of each gap is measured three times by sensor, and the average value is taken to determine intraobserver agreement. Soft tissue release was then undertaken if required based on the sensor data. Once the knee was well balanced, a fully cemented cruciate-retaining knee prosthesis was implanted in all cases.

The Knee Society Score (KSS) and the Western Ontario and McMaster Universities (WOMAC) were recorded preoperatively and postoperatively. The KSS score consists of a functional and clinical subscale, with both a maximum score of 100 points. The WOMAC score consists of pain, stiffness and physical function, 0 point is the best and 96 points is the worst.

Definitions of knee balance using the three methods:

1. SDA: moderate tension of the medial and lateral ligaments, and a side-to-side difference of no more than 2 mm at each knee flexion angle when inserting the trial implants.
2. GB: a gap difference between the medial and lateral compartments at 0° of knee flexion of less than 1 mm, and a balance of 1–4 mm larger on the lateral side than the medial side at 90° flexion. There was no femoral external rotation in the GII femoral prosthesis osteotomy, and the standard flexion gap was 2.5 mm wider on the lateral side than on the medial side.
3. Sensor: an intercompartmental pressure difference (ICPD) between the medial and lateral compartments of 30 N or less at each flexion angle. The pressure in the medial and lateral compartments was 0–100 N.

### Statistical analysis

The sensitivity of the SDA was calculated in comparison with the sensor data. The accuracy, sensitivity, specificity, and predictive values were calculated for the GB compared with the sensor data. Cohen’s kappa coefficient was calculated to determine the chance-corrected proportional agreement as slight (Cohen’s kappa coefficient 0.01–0.20), fair (Cohen’s kappa coefficient 0.21–0.40), moderate (Cohen’s kappa coefficient 0.41–0.60), substantial (Cohen’s kappa coefficient 0.61–0.80), and almost perfect (Cohen’s kappa coefficient 0.81–0.99) [7]. Statistical significance was set at *P* < 0.05.

Cumulative summation (CUSUM) is a sequential analysis method used to detect changes in performance and analyze the learning curve of a new technology [8]. CUSUM analysis was used to determine whether gap balance was achieved at 0°, 45°, 90°, and 120° flexion. CUSUM charts were created to display the CUSUM values against the number of procedures. The CUSUM graph decreases with each success, and a horizontal or downward-sloping line indicates an acceptable performance. Conversely, the CUSUM graph increases with each failure, and an upward-sloping line indicates an unacceptable performance. We used horizontal control lines to help interpret the acceptability of the performance. If the CUSUM line crossed the acceptable control line from above, this indicated good prior performance (GPP). If the CUSUM line crossed the unacceptable control line from below, this indicated poor prior performance (PPP) [9].

Statistical analysis was performed using SPSS 20 (IBM SPSS Statistics for Windows, version 22.0; IBM Corp., Armonk, NY).

## Results

Intraobserver variation of the sensor data assessed by calculating the intraclass correlation coefficient (ICC) was from 0.854 to 0.946. No statistically significant differences in agreement or reliability of intraobserver measurements were found.

### Sensitivity and compartment loads of SDA

In the 60 knees, the sensitivities of SDA compared with the sensor data at 0°, 45°, 90°, and 120° flexion were 63.3% (50.8–75.9%), 68.3% (56.2–80.5%), 80.0% (68.6–90.4%), and 80.0% (68.6–90.4%), respectively.

**Fig. 2:**
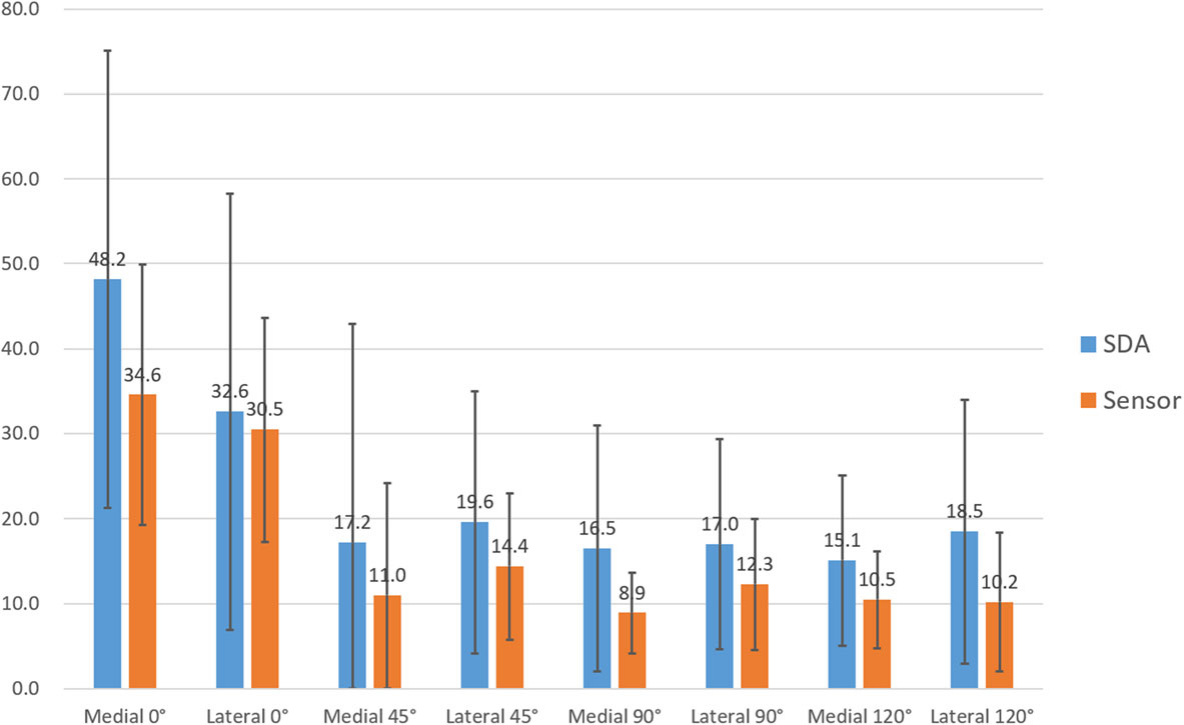
Compartmental pressure in the SDA group (60 knees) versus the sensor-balanced group (22 knees) at four knee flexion angles. SDA, surgeon-defined assessment

There was concordance between the SDA and sensor data at all four flexion positions in 22 knees, at 3 positions in 21 knees, at 2 positions in 10 knees, at 1 position in 4 knees, and at 0 positions in 3 knees. A comparison of all 60 knees with the group of 22 knees in which the sensor data showed soft tissue balance at all four positions showed that the compartment pressure was smaller in the sensor-balanced group than in the whole sample. The pressures in the medial and lateral compartments were significantly larger when the knee was in extension than flexion and decreased as the knee became more flexed (Fig. 2).

### Agreement between the GB and sensor data

The GB assessment was consistent with the sensor data in 37 knees at 0° and in 37 knees at 90°, while the GB and sensor data were inconsistent in 9 knees at 0° and in 6 knees at 90° (Table 1). The sensitivity of gap balancing was improved using the GB compared with SDA. Interestingly, the sensor data showed that the ICPD in knees balanced using the GB exceeded the value suggested by the manufacturer in some cases. The gap difference was larger than 1 mm (range 2–6 mm) at 0° in five knees, and larger than 4 mm (range − 5 to 0 or 6 mm) at 90° in eight knees.

**Table 1:** Consistency of the GB and sensor data

0°/90°	Sensor balance	Sensor unbalance
GB balance	37/37	9/9
GB unbalance	5/8	9/6

*GB* gap-balancer

Table 2 outlines the GB outcomes at each position. There were no differences between the GB measurements in extension and flexion. The accuracy, sensitivity, and positive predictive value of the GB measurements were higher than those of the SDA measurements. However, the specificity and negative predictive value of the GB measurements were not ideal. Cohen’s kappa coefficient for the accuracy of the GB measurements compared with the sensor data was 0.406 at 0° (indicating moderate agreement) and 0.227 at 90° (indicating fair agreement).

**Table 2:** Accuracy of gap-balancer measurements compared with sensor data at two knee flexion angles

Statistical measure of performance	0° mean (95% CI)	90° mean (95% CI)	*P* values (angle comparison)
Accuracy	76.7% (65.7% ~ 87.7%)	71.7% (59.9 ~ 83.4%)	0.536
Sensitivity	88.1% (75.0% ~ 94.8)	82.2% (68.7 ~ 90.7%)	0.448
Specificity	50.0% (29.0% ~ 70.9%)	40.0% (19.8 ~ 64.3%)	0.580
PPV	80.4% (68.5% ~ 92.4)	80.4% (68.5 ~ 92.4%)	1.000
NPV	64.2% (35.6 ~ 93.0)	42.9% (13.2 ~ 72.5%)	0.272

*CI* confidence interval, *PPV* positive predictive value, *NPV* negative predictive value

### CUSUM analysis

**Fig. 3:**
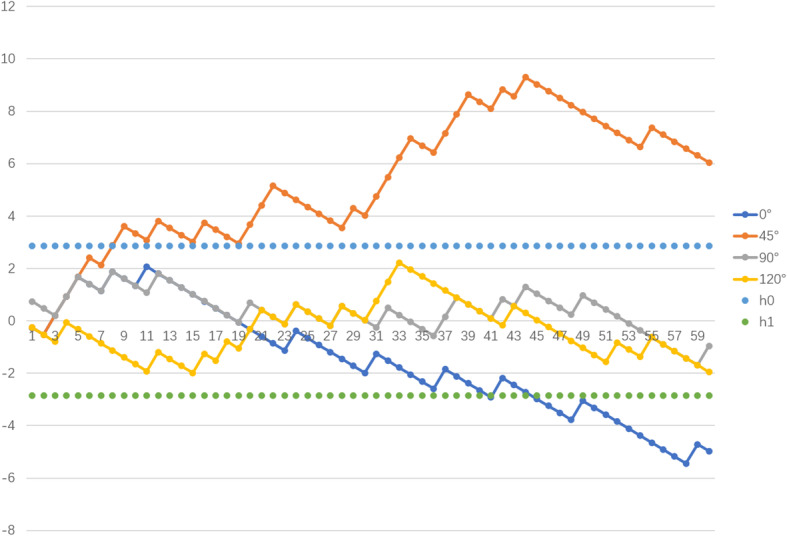
Combined continuous cumulative summation chart. h0, the unacceptable control line; h1, the acceptable control line

Figure 3 shows the learning curve for the sensor technology. The CUSUM at 0° flexion showed a downward trend from the 12th case onwards, although some cases were unbalanced; by case 45, the curve achieved GPP. The CUSUM at 90° and 120° showed successes and an occasional unbalanced downward trend from the 34th and 51st case onwards, respectively; however, these two curves never achieved GPP in all 60 cases. The CUSUM at 45° showed overall deterioration (upward slope), reaching PPP at case 8. Although there was a downward trend during this period, the curve continually fluctuated and did not fall below the line indicating PPP; the curve increased dramatically from the 28th case onwards, with attempts at correction after case 45 (downward slope).

### Clinic outcomes

The comparison of KSS scores and WOMAC scores of patients preoperatively and postoperatively is shown in Table 3. The average follow-up period was 12.6 months (6–22 months). The postoperative KSS scores and WOMAC scores were significantly improved compared with preoperative (*P* < 0.01).

**Table 3:** Comparison of KSS scores and WOMAC scores preoperatively and postoperatively

	Preoperative (mean ± SD)	Postoperative (mean ± SD)	*P* values
KSS functional score	66.6 ± 14.0	94.3 ± 6.8	< 0.01
KSS clinical score	49.3 ± 12.5	84.4 ± 13.9	< 0.01
KSS total score	116.0 ± 23.6	178.7 ± 19.2	< 0.01
WOMAC	47.2 ± 8.5	10.5 ± 7.7	< 0.01

## Discussion

TKA is a very successful orthopedic operation. However, although there is a relatively established system for TKA, about 20% of patients are not satisfied with the surgical outcome [2]. Soft tissue balancing helps to achieve a successful outcome. Surgeons have traditionally relied on SDA for soft tissue balancing, with few objective assessments. New electronic sensor technology provides real-time objective data intraoperatively to help determine the gap balance, giving a better TKA outcome [1, 10, 11]. However, it is unclear whether the gap balance achieved using SDA is improved by the use of a GB, and whether the sensor improves the SDA of gap balance (i.e., serves as a training tool for surgeons).

Regarding the soft tissue balance in TKA, previous studies mainly focused on the comparison of SDA and sensor data [6, 12]. MacDessi et al. [6] prospectively analyzed 322 patients undergoing TKA and demonstrated that the accuracy of SDA compared with the sensor was 63%, 57.5%, and 63.8% at 10°, 45°, and 90°, respectively, and that SDA had an overall sensitivity of 81% and specificity of 37.7%; they concluded that SDA is a poor predictor of the true soft tissue balance when compared with sensor data, particularly in assessing whether a knee is unbalanced. However, Elmallah et al. [12] compared 12 patients who underwent sensor-balanced TKA with 12 patients who underwent manual gap-balanced TKA by an experienced surgeon, and noted high rates of unbalanced knees that were not initially identified using the sensor-guided balance assessment.

In our study, the sensitivity of SDA was low at 0° because the surgeon aimed to acquire a stable knee and made the medial compartment slightly tighter than the lateral compartment. In addition, it was difficult to visualize the gap during valgus examination due to the presence of the patella and fat pad.

The present study showed that the pressures of the medial and lateral compartments after SDA were similar to those of the normal knee joint; that is, the ICPD was the largest at 0°, and the medial pressure was greater than the lateral pressure. As the knee was flexed, the pressure rapidly decreased. However, in the process of knee flexion from 45° to 120°, the pressure of the knee compartments tended to remain stable; that is, the pressure did not further decrease with the increase in the flexion angle.

Verstraete et al. [13] reported mean pressures of 114 N and 63 N, respectively, in the medial and lateral compartments at 0° flexion in eight non-arthritic cadavers; both the medial and lateral compartment pressures decreased rapidly from 0° to 10° knee flexion and stabilized after 20°, while the lateral pressure decreased slowly. The pressures in the medial and lateral compartments were only 30 N and 5 N at 90° flexion [13]. However, the absolute forces transmitted through the medial compartment were greater than the loads in the lateral compartment [13]. Risitano et al. [14] defined stable knees as those with a pressure of 50 ± 20 lb in the medial compartment, 35 ± 20 lb in the lateral compartment, and a mediolateral intercompartmental difference within 15 ± 5 lb, with the medial compartment slightly tighter than the lateral compartment.

Our study found that soft tissue balancing using the electronic sensor achieved a lower compartmental pressure compared with balancing performed using SDA alone. This may be related to the use of the GB and sensor to release the soft tissue again after SDA. Furthermore, in accordance with the sensor-assessed balance, we adjusted the pressure if it was greater than 100 N. Elmallah et al. [12] also found that the use of an electronic sensor resulted in additional soft tissue releases (outside of the surgeon’s standard releases), which resulted in lower intercompartmental pressures.

As far as we know, no previous study has compared the GB and electronic sensor data. The GB is different to SDA, as the GB achieves a constant opening force accurate to 1 mm. Thus, the GB is more accurate than SDA, especially at 0°. However, the specificity of the GB was relatively low, suggesting that the GB has a poor ability to detect unbalanced knees. Our study showed that the GB had good accuracy at 0°, but that the accuracy decreased with knee flexion, similarly to the accuracy of SDA. MacDessi et al. [6] reported that SDA was a poor predictor of knee imbalance, particularly as the knee flexion angle increases, with a sensitivity twice as large as the specificity.

In our study, some sensor-balanced knees had a larger gap difference between the medial and lateral compartments than that recommended by the manufacturer of the GB. This may be because the sensor only considers the ICPD. Therefore, although the interventricular difference was within the normal range, the medial and lateral pressure distribution did not conform to the physiological state. In a setting that emulates the surgical environment, an intact knee specimen under normal and native ligament tension creates a larger compressive load on the medial side than the lateral side [13], while the sensor does not consider this standard. To better simulate the characteristics of knee joint pressure distribution under physiological conditions, Risitano et al. [14] proposed that the balance standard of the electronic sensor should have a greater pressure in the medial compartment than the lateral compartment; that is, a medial minus lateral pressure of 10–20 lb, rather than the commonly used pressure difference of ± 15 lb. According to our data, the pressure was 10–40 N greater in the medial compartment than the lateral compartment at 0°.

As a novel technology, the electronic sensor has a learning curve. Surgeons need to become proficient in using the sensor to adapt to the new technology. Training objectives include minimizing the operation time and accurately adjusting the unbalanced pressure. Lakra et al. [15] analyzed 287 consecutive patients who underwent TKA using an electronic sensor to achieve gap balance. It took approximately 41 cases of sensor-assisted TKA to reduce the operation time of the sensor-based surgery from 120 min to 109 min, which was close to the conventional operation time [15]. This suggests that surgeons master the use of sensor technology after 41 TKA cases. Woon et al. [9] used CUSUM to evaluate the learning curve of TKA with an electronic sensor. The data of 10°, 45°, and 90° were analyzed. During the non-blinded Phase I, the CUSUM 10° reached GPP at case 41, the CUSUM 90° showed a downward trend but never achieved GPP, and the CUSUM 45° achieved PPP at case 11 [9]. However, MacDessi et al. [16] used a chi-squared learning curve histogram and found that the capacity of SDA to determine balance compared with sensor data did not improve with ongoing use of the sensor.

Our results showed that the learning curve for the sensor technology was the shortest at 0°, followed by 90° and 120°, while 45° was the most difficult, which was similar to the findings of Woon et al. [9]. This may be because the extension balance of TKA is more important than the flexion balance. In TKA, the surgeon should first assess the extension gap balance to ensure excellent lower limb alignment, then assess the gap balance at 90° flexion, and finally assess the balance at 45° and 120°. In addition, the soft tissue balance at different knee flexion angles affects the balance at other angles; the soft tissue release at one angle sometimes leads to a change in the ICPD at another angle. In such cases, surgeons may need to compromise and prioritize the balancing of the extension gap.

The present study has some limitations. First, the sample size was relatively small, especially in the CUSUM analysis. While post hoc sample size estimation showed: according to the 76.6% agreement between GB and sensor group, the allowable error is 10%. The 95% CI of the agreement rate was estimated by PASS 14.0, and 77 knees needed to be studied. The sample size of this study is 60 knees, while the gap is acceptable. It may have been better to cross the learning curve at 90° and 120° in the later cases. Second, the acceptable limits of the ICPD are not clear. Previous research has showed that a maximum ICPD of 15 psi may be an inappropriate definition of knee balance, and that the ICPD should be 0–40 psi [6, 17]. Furthermore, Meneghini et al. [18] showed that the intercompartmental force difference did not support the categorical mediolateral force difference of ≤ 15 lb. In knees balanced using the sensor, we found a similar compartmental pressure to that reported by Verstraete et al. [13]. Our study defined knee balance as an ICPD of 30 N at each flexion angle, which is the standard provided by the sensor manufacturer. However, the optimal target ligament balance for each patient undergoing TKA remains unknown. Future studies are required to evaluate the differences in functional outcomes between these patient cohorts and to assess the role of different load measurements [16]. Third, we conducted early period follow-up on the postoperative function of the patients. It is obvious that the knee functions after TKA are significantly improved. However, our study did not compare with the postoperative follow-up of non-sensor groups, which is also a limitation of our study. Fourth, we only included one surgeon, and its advantage is to avoid the errors caused by surgical techniques and measurement methods. However, we cannot effectively count the differences between surgeons and measured the interclass correlation coefficient

## Conclusion

In the present study, SDA was a poor predictor of knee balance. The use of a GB improved the accuracy of gap balancing, but there was still room for improvement, particularly when assessing knees that were unbalanced. SDA showed a learning effect with the ongoing use of the sensor at all knee positions except 45°, which was the most difficult position to balance. Although the sensor is the gold standard, the optimal ICPD for each patient undergoing TKA remains unknown. Further study is necessary to compare the outcome among the patient cohorts with different ICPDs.

## Data Availability

The datasets obtained and/or analyzed during the current study are available from the corresponding author on reasonable request.

## Abbreviations

TKA: Total knee arthroplasty
SDA: Surgeon-defined assessment
GB: Gap-balancer
CUSUM: Cumulative summation
GPP: Good prior performance
PPP: Poor prior performance
